# Disseminated Well-Differentiated Gastro-Entero-Pancreatic Tumors Are Associated with Metabolic Syndrome

**DOI:** 10.3390/jcm8091479

**Published:** 2019-09-17

**Authors:** Ana P. Santos, Clara Castro, Luís Antunes, Rui Henrique, M. Helena Cardoso, Mariana P. Monteiro

**Affiliations:** 1 Department of Endocrinology, Portuguese Oncology Institute of Porto (IPO Porto), 4200-072 Porto, Portugal; 2 Clinical Research Unit, Research Center of IPO Porto (CI-IPOP), Portuguese Oncology Institute of Porto (IPO Porto), 4200-072 Porto, Portugal; 3 Cancer Epidemiology Group, Research Centre of IPO Porto (CI-IPOP), Portuguese Oncology Institute of Porto (IPO Porto), 4200-072 Porto, Portugal; clara.castro@ipoporto.min-saude.pt (C.C.); luis.antunes@ipoporto.min-saude.pt (L.A.); 4 EpiUnit, Institute of Public Health, University of Porto, 4050-600 Porto, Portugal; 5 Department of Pathology, Portuguese Oncology Institute of Porto (IPO Porto), 4200-072 Porto, Portugal; rmhenrique@icbas.up.pt; 6 Cancer Biology and Epigenetics Group, Research Centre of IPO Porto (CI-IPOP), Portuguese Oncology Institute of Porto (IPO Porto), 4200-072 Porto, Portugal; 7 Department of Pathology and Molecular Immunology, Institute of Biomedical Sciences Abel Salazar (ICBAS), University of Porto, 4050-600 Porto, Portugal; 8 Clinical and Experimental Endocrinology, Unit for Multidisciplinary Research in Biomedicine, UMIB-ICBAS, University of Porto, 4050-313 Porto, Portugal; helenacardoso@icbas.up.pt; 9 Department of Endocrinology, Centro Hospitalar Universitário do Porto (CHUP), 4099-028 Porto, Portugal; 10 Department of Anatomy, Institute of Biomedical Sciences Abel Salazar (ICBAS), University of Porto, 4050-313 Porto, Portugal; mpmonteiro@icbas.up.pt

**Keywords:** gastro-entero-pancreatic neuroendocrine tumor, metabolic syndrome, visceral obesity, fasting glucose abnormalities

## Abstract

The association of well-differentiated gastro-entero-pancreatic neuroendocrine tumors (WD GEP-NETs) with metabolic syndrome (MetS), abdominal obesity, and fasting glucose abnormalities was recently described. The aim of this study was to evaluate whether the presence of MetS or any MetS individual component was also influenced by GEP-NET characteristics at diagnosis. A cohort of patients with WD GEP-NETs (*n* = 134), classified according to primary tumor location (gastrointestinal or pancreatic), pathological grading (G1 (Ki67 ≤ 2%) and G2 (>3 ≤ 20%) (WHO 2010), disease extension (localized, loco-regional, and metastatic), and presence of hormonal secretion syndrome (functioning/non-functioning), was evaluated for the presence of MetS criteria. After adjustment for age and gender, the odds of having MetS was significantly higher for patients with WD GEP-NET grade G1 (OR 4.35 95%CI 1.30–14.53) and disseminated disease (OR 4.52 95%CI 1.44–14.15). GEP-NET primary tumor location or secretory syndrome did not influence the risk for MetS. None of the tumor characteristics evaluated were associated with body mass index, fasting plasma glucose category, or any of the individual MetS components. Patients with GEP-NET and MetS depicted a higher risk of presenting a lower tumor grade and disseminated disease. The positive association between MetS and GEP-NET characteristics further highlights the potential link between the two conditions.

## 1. Introduction

Gastro-entero-pancreatic neuroendocrine tumors (GEP-NETs) were previously a rare entity before the 6.5-fold increase in incidence observed over the past four decades [1]. As a matter of fact, GEP-NETs are now the second most frequent digestive tumors after colorectal adenocarcinoma [2]. The reasons underlying the exponential increase in the incidence of sporadic GEP-NETs remain largely unknown, even though significant advances toward understanding the genetics and molecular mechanisms associated with GEP-NETs biology were made [3].

One of the most remarkable achievements of oncology in the 21st century was the finding that most cancers could be preventable diseases [4]. The association of environmental factors with tumor development, disease recurrence, and mortality risks were demonstrated by a large number of studies for several different types of cancers [5,6,7,8]. In particular, obesity, metabolic syndrome (MetS), and type 2 diabetes mellitus (T2DM), which are also experiencing an exponential rise worldwide, have been implicated as risk factors for cancer incidence and disease recurrence [9,10]. Despite the available evidence that those metabolic conditions are risk factors for several different tumor types, the amount of data available concerning GEP-NETs is more limited. The association between well-differentiated (WD) GEP-NETs with MetS and some of the MetS individual components, namely abdominal obesity and abnormal fasting plasma glucose (FPG), was recently described by our group [11].

The aim of the current study was to evaluate whether the presence of MetS and individual MetS components at the time of WD GEP-NET diagnosis was associated with any specific tumor characteristics, such as grading, staging, primary tumor location, or hormonal hypersecretion, that were likely to influence the tumor biological behavior and disease prognosis.

## 2. Experimental Section

Patients with confirmed WD GEP-NETs were recruited from the Endocrine Tumors Clinic of a single large tertiary referral center for oncologic diseases (Portuguese Oncology Institute of Porto (IPO P)). The inclusion criteria included having a confirmed diagnosis of WD GEP-NETs by histopathology and/or PET-68Ga-DOTA-NOC. Patients excluded from the study were those who were younger than 18 years old when first diagnosed, as well as those harboring familial GEP-NETs, neuroendocrine carcinoma (NEC), and/or a type 1 gastric endocrine tumor (T1-GET), as these tumors are recognized as having a distinctive and well-established etiology and biological behavior [12].

From the patients with confirmed WD GEP-NETs that consented to participate in the study (*n* = 159), those who did not fulfill the inclusion criteria or had insufficient data for analysis were excluded, while the remaining eligible patients were included in the study for statistical analysis (*n* = 136). Tumors were classified according to primary tumor location: gastrointestinal (GI-NET) or pancreatic (pNET); functioning or non-functioning (F or non-F); pathological WHO grading into G1 (<2 mitotic count; Ki-67 index ≤ 2) and G2 (2–20 mitotic count; Ki-67 index 3–20) and disease extension (localized, loco-regional, and disseminated) [13]. Disease extension was categorized as localized, locoregional, or disseminated, to enable the grouping of WD GEP-NETs, since ENETS staging categories, depending on the primary tumor location, diverge. Patients with insufficient data to allow for grading were classified as WD GEP-NET if found to express somatostatin receptors on PET-^68^Ga-DOTA-NOC (*n* = 6). Patients with WD GEP-NETs metastatic tumors and carcinoid syndrome without any visible pancreatic or thoracic lesions on imaging studies were assumed as midgut primary tumor (*n* = 2). No insulinoma or rare functional pancreatic NET presenting with hyperglycemia, such as glucagonoma, VIPoma, or somatostatinoma, were included in this series [14].

Patients with WD GEP-NETs were assessed for body mass index (BMI) class [15], fasting plasma glucose (FPG) category [16], and the presence or absence of MetS diagnostic criteria or any individual MetS component [17].

Data for analysis were collected during face-to-face patient interviews, to assess past medical history of T2DM, hypertension, dyslipidemia, ongoing medications, and family history of T2DM. Anthropometric parameters, such as height, weight, waist circumference (WC), and blood pressure (BP) were measured during the study visit. Additionally, biochemical data, including FPG and lipid profile, were evaluated after blood sampling in our institution for treatment-naïve patients, or retrospectively through data-files recollection of parameters before initiation of any treatment intervention at the referring healthcare institutions, whenever the patient was already under pharmacological treatment when first observed at our center.

Patients were classified into three categories according to BMI: normal weight (BMI < 25 kg/m^2^), overweight (BMI 25–29.9 kg/m^2^), or obese (BMI ≥ 30 kg/m^2^) [15]. They were also classified according to FPG levels: normoglycemic (NG; FPG < 100 mg/dL), impaired fasting glucose (IFG; FPG ≥ 100 < 126 mg/dL), or T2DM (T2DM; FPG ≥ 126 mg/dL) [16]. MetS was classified according to the Joint Interim Statement (JIS) of IDFTFEP (International Diabetes Federation TaskForce on Epidemiology and Prevention)/NHLBI (National Heart, Lung, and Blood Institute)/AHA (American Heart Association)/WHF (World Heart Federation)/IAS (International Atherosclerosis Society) /IASO (International Association for the Study of Obesity) criteria [17]: WC ≥ 88 cm (female) or 102 cm (male); systolic BP ≥ 130 or diastolic BP ≥ 85 mmHg or previous history of high BP or under BP-lowering medication; HDL-cholesterol (HDL-c) < 40 mg/dL (male) or ≤ 50 mg/dL (female) or drug treatment to reduce HDL-c; triglycerides (TG) ≥150 mg/dL or under triglyceride-lowering drugs; FPG ≥ 100 mg/dL or ongoing treatment with glucose-lowering drugs.

This study was approved by the National Data Protection Committee (CNPD /4906/2015) and Institutional Ethics Review Board (IPOP/366/2013). All participants provided informed consent prior to study enrolment.

Statistical analysis was performed using IBM SPSS Statistics version 24.0 (IBM, New York, USA). Categorical and continuous variables were summarized using descriptive statistics (frequencies for categorical; mean/standard deviation or median/interquartile range for continuous, as appropriate). Proportions were compared using the Chi-square or Fisher’s exact test, as appropriate. Means were compared using Student’s *t* test or ANOVA, while medians were compared using the Mann–Whitney or Kruskal–Wallis tests.

A backward stepwise (Wald) method was used to obtain a multivariable logistic regression model, using the patient and tumor characteristics (sex, age at diagnosis, tumor primary site, grading, stage, and clinical hypersecretion syndrome). A level of significance of 0.05 was adopted.

## 3. Results

The cohort of patients with WD GEP-NETs (*n* = 134) was divided into two groups, according to baseline characteristics and considering the absence (*n* = 57) or presence of MetS (*n* = 77) at the time of tumor diagnosis (Table 1). Patients in the group with MetS were predominantly male (*p* = 0.014), older (*p* < 0.001), and had a higher BMI at diagnosis (*p* < 0.001). When comparing the two patient groups, there was a homogeneous distribution in terms of primary tumor location (*p* = 0.652), presence of hormonal secretion syndrome (*p* = 0.187), and metastatic disease (*p* = 0.104). Grade 1 (G1) tumors were found to be more frequent in the MetS group; although, the difference was not statistically significant (*p* = 0.076) (Table 1).

**Figure 1:**
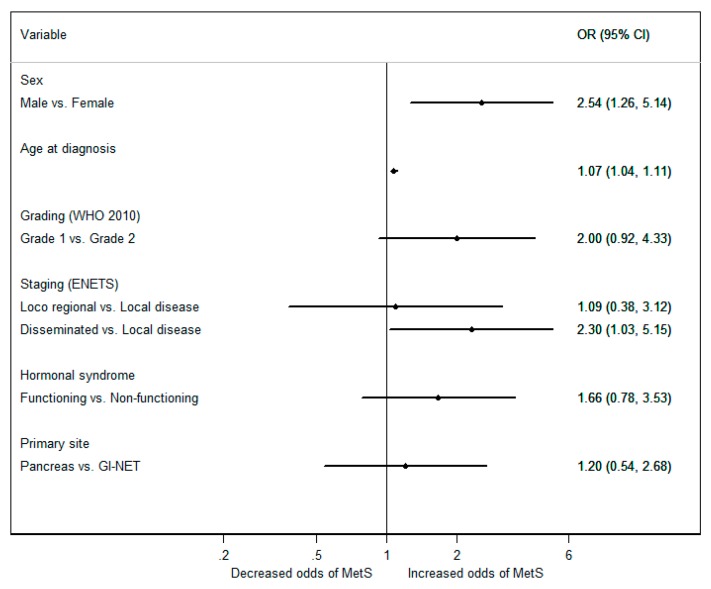
Odds ratios (ORs) and 95% confidence intervals (CIs) for the occurrence of metabolic syndrome, according to the characteristics of patients with WD GEP-NETs, using a univariate logistic regression.

**Figure 2:**
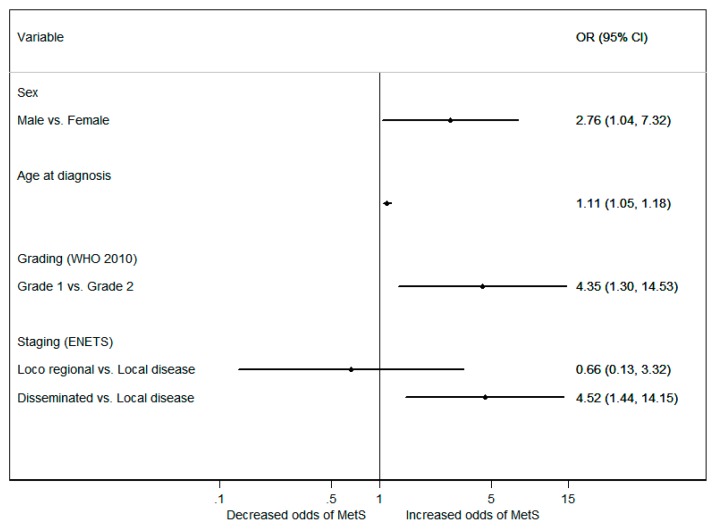
Odds ratios (ORs) and 95% confidence intervals (CIs) for the occurrence of metabolic syndrome, according to the characteristics of patients with WD GEP-NETs, using a multivariate logistic regression.

The odds of patients with WD GEP-NETs having MetS was significantly higher in males (*p* = 0.009) and increased with age (*p* < 0.001) (Figure 1). After adjusting for age and gender, the positive association between disseminated disease and MetS persisted, with patients with metastatic disease depicting odds of having MetS over four times greater than patients with localized disease (OR 4.52 95%CI 1.44–14.15; *p* = 0.010). In addition, G1 grade was found to be significantly associated with MetS (GEP-NETs G2 vs. G1; (OR 4.35 95%CI 1.30–14.53; *p* = 0.018), while the primary tumor location or hormonal secretory status of GEP-NETs did not influence the risk of MetS (Figure 2).

No significant association was found between the primary tumor location of WD GEP-NETs, the presence of hormonal secretion syndrome, the tumor grading or disease extension, and the presence of any of the individual MetS components at diagnosis (Table 2), or between the WD GEP-NET characteristics and BMI or FPG classification (Table 3).

## 4. Discussion

GEP-NETs are a group of heterogeneous neoplasms that may present considerable differences in what concerns primary tumor location, pattern of hormone secretion, proliferative behavior, and disease extension at diagnosis. Obesity, MetS, and T2DM were recognized as risk factors for several cancers [5,9,18]. These include esophageal, pancreatic, colorectal, endometrial, kidney, and breast cancer in post-menopausal women [5,18,19,20,21,22,23,24]. However, whether any of these metabolic conditions are also risk factors for GEP-NETs or are able to negatively influence disease behavior is yet to be fully established. Notwithstanding, our group has shown in a case-control study that WD GEP-NETs are associated with visceral obesity, elevated FPG, and MetS [11]. Given these prior findings, our current aim was to investigate whether there were any further associations between the pathological features of WD GEP-NETs and the anthropometric and clinical parameters that characterize MetS.

The incidence of GEP-NETs increased over the last four decades, disclosing a current prevalence of 6.4 cases/100,000 inhabitants [1,2]. The upsurge in GEP-NETs was initially attributed to improved medical skills, which led to an increased rate of incidental diagnosis by the widespread use of imaging techniques, while the search for other possible mechanisms underlying the unprecedented disease burden did not attract extensive investigation. Still, epidemiological data derived from several national registries suggest that both genetic and environmental factors must be involved in the phenomenon, explaining the ethnic and geographical differences observed in GEP-NET patterns [25]. Nevertheless, most studies that were aimed at unravelling the biology of GEP-NETs focused primarily on tumor genetics or molecular pathways underlying intrinsic pathological features [3,26,27], while the potential contribution of environmental factors was mostly neglected. Indeed, only a small number of retrospective studies have addressed the potential relationship between obesity, MetS, or T2DM and GEP-NETs [26,28,29], and the rare studies available were predominantly dedicated to pNETs only [30,31]. In 2016, the largest subset meta-analysis ever performed disclosed BMI and T2DM, in addition to family history of cancer, as unpredicted risk factors for stomach, pancreas, and small-intestine GEP-NETs [32]. Furthermore, visceral obesity, high plasma triglycerides, abnormal FPG, and MetS were found to be associated with an increased risk of WD GEP-NETs in a case-control study performed by our group [11]. Previously, MetS was identified only as a risk factor for a subgroup of rectal WD GEP-NETs by two independent studies conducted in South Korea [33,34].

The core pathological feature that characterizes MetS is hyperinsulinism. In turn, hyperinsulinism leads to the subsequent activation of the insulin-IGF1 axis that has been theoretically proposed to support the relevance of MetS for WD GEP-NET biology [35]. Consequently, the use of insulin-sensitizing agents able to mitigate hyperinsulinism, such as metformin, for the prevention and treatment of cancer was also suggested. Indeed, the potential benefits of metformin as an anticancer drug are supported not only by several in vitro and in vivo experimental studies [36], but also by human data derived from epidemiological studies and prospective clinical trials [37]. Nevertheless, the proposed mechanisms responsible for the anticancer effects of metformin are not only limited to the improvement of insulin sensitivity, decreased hyperinsulinism, and the inhibition of the insulin-IGF1 axis, but also other potential direct actions, such as inhibiting the AMPK/Akt/PI3K mTor pathway and enhancing CD8^+^ T cells, which are key players in mediating immunity to tumors, for immune-mediator anticancer effects [38]. Indeed, the inhibition of the AMPK/Akt/PI3K mTor pathway is a well-known target for NETSs therapy, with everolimus being approved for the treatment of metastatic unresectable WD pNETs [39].

Our study aimed to evaluate the association of the four main characteristics of WD GEP-NETs, namely primary tumor location, presence of hypersecretion syndrome, WHO grade, and stage, with the occurrence of MetS. We were able to demonstrate, for the first time, that patients with WD GEP-NETs and MetS, independent of age or gender, are more likely to have lower-grade tumors or present advanced-stage disease at diagnosis. In fact, despite the fact that patients with MetS were more likely to be older, in parallel to what is observed in the general background population, this parameter was not shown to influence WD GEP-NET characteristics [40]. Moreover, neither the WD primary tumor location of GEP-NETs nor the presence of hormonal secretion syndrome was associated with MetS or any of the individual components of MetS. Furthermore, although metabolic alterations are usually associated with functioning and non-functioning pNETs, in this cohort, functioning GI-NETs with carcinoid syndrome were also shown to be associated with MetS in 41.8% of the cases. In fact, a considerable number of subjects in our cohort had small-intestinal WD GEP-NETs that, despite presenting small-size primary tumors, were often found to be metastatic at diagnosis. Of particular note is the fact that more than half of the patients with metastatic disease also had MetS features. This observation raises the need to investigate the impact of WD GEP-NETs on MetS, as the mechanistic reasons for this observation are not entirely clear and thus warrant further investigation. Notwithstanding the widespread dissemination of the disease, patients with GEP-NETs usually preserve an overall very good health status, with rare cases of cancer cachexia, which is particularly notorious in patients with GI-NETs, as confirmed by the nearly two-thirds of patients with an overweight or obesity BMI grade.

One of the main strengths of this study is that it enrolled a reasonably large patient cohort for what is considered a relatively rare disease, along with consistent data retrieval, since all clinical and anthropometrical parameters were assessed by a single clinical researcher. However, some limitations must also be acknowledged. First, this study was conducted in a single center; therefore, and despite the sample size, these results require further validation, ideally in multicenter prospective studies. In addition, since our study was conducted in an end-of-line tertiary center, a small proportion of patients with WD GEP-NETs were already under pharmacological treatment for the disease when referred and first evaluated at our center. For these cases, clinical parameters were obtained retrospectively to minimize any possible bias for statistical analysis concerning data before treatment initiation. Last, but not least, other potential confounding factors, such as family history of cancer, cigarette smoking, alcohol consumption, dietary habits, physical activity, occupation, and socioeconomic status, were not evaluated in this study; therefore, we are unable to estimate whether these could have had any impact on the study results.

Overall, our data emphasize the unmet need to further explore the mechanisms underlying the association of obesity, abdominal obesity, and the metabolic abnormalities that characterize MetS with GEP-NETs, as such an exploration could not only improve the knowledge of the causes for the recently increased burden of these tumors, but it could also open a field of work that might lead to the disclosure of novel and more effective preventive and treatment avenues, as already described for other types of cancer.

In conclusion, this study demonstrates, for the first time, a positive association of MetS with WD GEP-NETs disease extension and tumor grade. Our results demonstrate that patients with WD GEP-NETs and MetS are more likely to have tumors with better differentiation and disseminated disease at diagnosis, independent of primary tumor location and hormonal status. Our findings suggest that further research on the mechanisms underlying the metabolic abnormalities associated with WD-GEP-NETs is warranted, as these underlying mechanisms could potentially harbor the keys for novel and more effective preventive and treatment interventions.

## Figures and Tables

**Table 1:** General patient and well-differentiated gastro-entero-pancreatic neuroendocrine tumor (WD GEP-NET) characteristics (*n* = 134) in the two patient groups, according to the presence of metabolic syndrome diagnostic criteria.

WD GEP-NETs	Without MetS (*n* = 57)	With MetS (*n* = 77)	*p*
Gender-*n* (%)	21 (36.8) M/36 (63.2) F	46 (59.7) M/31 (40.3) F	0.009
Age-Mean (min.-max.)	57.2 (30–78) y	65.9 (42–85) y	<0.001
Age at Diagnosis (min.-max.)	53.9 (29–78) y	62.4 (38–85) y	<0.001
Weight (kg)-Mean ± SD	65.7 ± 11.4	76.4 ± 12.8	<0.001
BMI (kg/m^2^)-Median (IQR)	24.3 (4.05)	27.8 (5.47)	<0.001
WC (cm)-Mean ± SD	87.5 ± 10.6	99.3 (10.5)	<0.001
SBP (mmHg)-Mean ± SD	127.8 ± 14.6	140.7 ± 22.1	<0.001
DBP (mmHg)-Mean ± SD	72.9 ± 10.2	75.2 ± 12.4	0.262
HDL-c (mg/dL)-Mean ± SD	55.5 ± 13.2	46.6 ± 11.0	<0.001
Triglycerides (mg/dL)-Median IQR)	99.0 (13.0)	137.0 (83.5)	<0.001
FPG (mg/dL)-Median (IQR)	92.0 (13.0)	109.0 (18.5)	<0.001
Primary Tumor Location (*n* = 131)			0.652
GI-NET	43 (76.8)	55 (73.3)	
pNET	13 (23.2)	20 (26.7)	
Hormonal Syndrome (*n* = 119)			0.187
Functioning *	17 (32.1)	36 (67.9)	
Non-Functioning	29 (43.9)	37 (56.1)	
2010 WHO Gradinge (*n* = 127) ^#^			0.076
Grade 1	34 (61.8)	55 (76.4)	
Grade 2	21 (38.2)	17 (23.6)	
Staging (*n* = 122)			0.104
Localized Disease	24 (46.2)	22 (31.4)	
Locoregional Disease	10 (59.2)	10 (14.3)	
Metastatic Disease	18 (34.6)	38 (54.3)	
Extra-Hepatic Metastatic Disease ^ς^	5 (26.3)	8 (21.1)	0.448
Neuroendocrine Tumors pt. Treatments (*n* = 134)			
Surgery *-*n* (%)Liver Ablative Therapies–*n* (%)	10 (17.5)	20 (26.0)	0.298
Somatostatin Analogues–*n* (%)	35 (45.5)	42 (54.5)	0.383
Target Therapies–*n* (%)	-	-	-
PRRT–*n* (%)	5(8.8)	4 (5.2)	0.495
Chemotherapy–*n* (%)	1(1.8)	1 (1.3)	1.000

WD GEP-NETs: well-differentiated gastro-entero-pancreatic neuroendocrine tumors; MetS: metabolic syndrome; BMI: body mass index; WC: waist circumference; SBP: systolic blood pressure; DBP: diastolic blood pressure; FPG: fasting plasma glucose; GI-NET: gastrointestinal neuroendocrine tumor; pNET: pancreatic neuroendocrine tumor; WHO: World Health Organization; ENETS: European Neuroendocrine Tumor Society. * 49/119 (41.8%) patients with carcinoid syndrome (33 patients with MetS and 13 patients without MetS) and 2/119 (1.7%) patients with sporadic gastrinoma (100% with MetS)). ^#^ WHO 2010 Grade was used since 2013 and was the date of first patient enrolment, ^ς^ 3/13 bone metastasis; 8/13 peritoneal implants and 2/13 other locations.

**Table 2:** Presence of the metabolic syndrome individual components in patients with WD GEP-NETs, according to tumor characteristics (*n* = 134).

	Abdominal Obesity		Hypertension		Low HDL-c		High TG		High FPG	
	*n* (%)	*p*	*n* (%)	*p*	*n* (%)	*p*	*n* (%)	*p*	*n* (%)	*p*
Primary Tumor Location (*n* = 133)		0.536		0.084		0.803		0.384		0.194
GI-NET	47 (51.6)		69 (70.4)		51 (52.0)		35 (35.7)		52 (53.1)	
pNET	18 (58.1)		19 (54.3)		18 (54.5)		15 (44.1)		21 (60.0)	
Hormonal Syndrome (*n* = 121)		0.430		0.268		0.430		0.507		0.673
Functioning	28 (58.3)		39 (72.2)		34 (63.0)		22 (40.7)		31 (57.4)	
Non-Functioning	32 (50.8)		42 (62.7)		30 (45.5)		23 (34.8)		41 (61.2)	
WHO Grade (*n* = 129)		0.648		0.178		0.601		0.978		0.515
Grade 1	41 (48.8)		62 (69.7)		49 (55.1)		34 (38.2)		50 (56.2)	
Grade 2	18 (52.9)		23 (57.5)		19 (50.0)		15 (38.59		20 (50.2)	
ENETS Staging (*n* = 124)		0.633		0.677		0.092		0.336		0.194
Localized Disease	24 (46.2)		30 (65.2)		20 (44.4)		16 (34.8)		24 (52.2)	
Locoregional Disease	10 (59.2)		13 (65.0)		11 (55.0)		7 (35.0)		8 (40.0)	
Metastatic Disease	18 (34.6)		40 (69.0)		35 861.4)		25 (43.9)		37 (63.8)	

WD GEP-NETs: well-differentiated gastro-entero-pancreatic neuroendocrine tumors; MetS: metabolic syndrome; GI-NET: gastrointestinal neuroendocrine tumor; pNET: pancreatic neuroendocrine tumor; WHO: World Health Organization; ENETS: European Neuroendocrine Tumor Society.

**Table 3:** Association of WD GEP-NET characteristics with the BMI grade and fasting plasma glucose (FPG) classification at diagnosis.

WD GEP-NETs	BMI Grade				BMI Grade			
	Normal	Overweight	Obesity	*P*	Normal	AFPG	T2DM	*p*
Primary Tumor Location (*n* = 132)				0.187				0.326
GI-NET	31 (64.6)	42 (76.4)	24 (82.8)		59 (74.7)	22 (81.5)	17 (63.0)	
pNET	17 (35.4)	13 (26.3)	5 (17.2)		20 (25.3)	5 (18.5)	10 (37.0)	
Hormonal Syndrome (*n* = 120)				0.281				0.281
Functioning	28 (63.6)	23 (46.9)	15 (55.6)		36 (53.7)	11 (45.8)	20 (66.7)	
Non-Functioning	16 (36.4)	26 (53.1)	12 (44.4)		31 (46.3)	13 (54.2)	10 (33.3)	
WHO Grade (*n* = 129)				0.622				0.698
Grade 1	17 (36.2)	16 (29.6)	7 (25.9)		20 (34.2)	7 (26.9)	7 (25.9)	
Grade 2	30 (63.8)	38 (70.4)	20 (74.1)		50 (65.8)	19 (73.1)	20 (74.1)	
ENETS Staging (*n* = 124)				0.234				0.251
Localized Disease	17 (39.5)	18 (33.3)	10 (38.5)		29 (39.7)	9 (34.6)	8 (32.0)	
Locoregional Disease	9 (20.9)	5 (9.3)	6 (23.1)		14 (19.2)	1 (3.8)	5 (20.0)	
Metastatic Disease	17 (	31 (57.4)	10 (38.5)		30 (41.1)	16 (61.5)	12 (48.0)	

WD GEP-NETs: well-differentiated gastro-entero-pancreatic neuroendocrine tumors; MetS: metabolic syndrome; GI-NET: gastrointestinal neuroendocrine tumor; pNET: pancreatic neuroendocrine tumor; WHO: World Health Organization; ENETS: European Neuroendocrine Tumor Society.
